# Visualization of Trochlear Dysplasia Using 3-Dimensional Curvature Analysis in Patients With Patellar Instability Facilitates Understanding and Improves the Reliability of the Entry Point to Trochlea Groove Angle

**DOI:** 10.1016/j.asmr.2024.101010

**Published:** 2024-09-26

**Authors:** Johannes M. Sieberer, Nancy Park, Armita R. Manafzadeh, Shelby T. Desroches, Kelsey Brennan, Curtis McDonald, Steven M. Tommasini, Daniel H. Wiznia, John P. Fulkerson

**Affiliations:** a Department of Orthopaedics and Rehabilitation, Yale School of Medicine, New Haven, Connecticut, U.S.A.; b Yale Institute for Biospheric Studies, Yale University, New Haven, Connecticut, U.S.A.; c Department of Statistics and Data Science, Yale University, New Haven, Connecticut, U.S.A.; d Department of Mechanical Engineering and Material Science, Yale School of Engineering and Applied Science, New Haven, Connecticut, U.S.A.

## Abstract

**Purpose:**

To examine a method to visualize a 3-dimensional (3D) rendered distal femur using 3D curvature analysis and to compare models of patellofemoral instability (PFI) with controls to study the reliability of the entry point to trochlear groove angle (EPTG) metric.

**Methods:**

The 3D models of patients with recurrent patellar instability, defined by at least 2 reported patellar dislocation events, and age- and sex-matched controls were created from computed tomography scans. Curvature was calculated to highlight the proximal trochlear ridges and the trochlear groove by overlaying them on the 3D models. Anteroposterior views with and without curvature visualization were created and used for qualitative comparison and to measure the EPTG. The EPTG was measured by 2 raters with and without the aid of the curvature maps. Significant differences between patients with PFI and controls were compared with a Mann-Whitney *U* test. Inter-rater reliability was calculated using interclass correlation coefficients, classified according to literature and compared using a permutation test. Significance was assumed at .05.

**Results:**

Qualitive analysis between 30 PFI patient knees (age: 23.9 ± 8.4 years, female/male: 24/6) and 30 control knees (age: 21.8 ± 5.6 years, female/male: 22/8) showed that in general, patients with PFI have a lateralized medial ridge and trochlear groove, with the trochlear groove being shorter and shallower. Qualitatively, differences between patients with PFI and controls were significant for measurements both with and without the aid of the curvature maps. Inter-rater reliability was significantly (*P* = .0349) better when using the curvature visualization.

**Conclusions:**

Curvature-based visualization aids overlain on a 3D model have the power to increase the information gained from 3D imaging and corresponding 3D models, amplifying their potential value in clinical decision-making. Such visualizations facilitate both the identification of qualitative differences between patient and control morphology and improve the reliability of the EPTG trochlear dysplasia metric.

**Level of Evidence:**

Level III, retrospective cohort study.

The femoral trochlea guides and stabilizes the patella along its tracking path. In cases of trochlear dysplasia, which is a risk factor for patellar instability, the function of the trochlea to guide the patella is either reduced or lost.^1^, ^2^, ^3^ Therefore, the shape of the trochlea must be considered when evaluating a patient’s risk of instability. Traditionally, dysplasia has been diagnosed using sagittal and axial x-rays and axial slices of 3-dimensional (3D) imaging modalities such as magnetic resonance imaging and computed tomography (CT).^3^^,^^4^

However, methods of visualizing and quantifying trochlear morphology based on these 2-dimensional (2D) images only provide a limited understanding of the complex 3D shape of the trochlea and its impact on tracking. For example, one 2D classification uses the most proximal axial slice of the trochlear groove, leading to limited understanding of its complex shape.^2^ In addition, consistently selecting the same slice remains a challenge, leading to low inter-rater reliability.^5^ A similar issue also exists with other quantitative 2D metrics, such as the sulcus angle.^6^

The 3D renderings and physical models are better at comprehensively capturing this unique shape.^1^^,^^2^^,^^7^ The 3D printed models can highlight unique features and facilitate tactile interaction, but their use is limited due to high cost and lack of widespread infrastructure to support routine 3D printing, such as equipment and dedicated staff. The 3D computer renderings, while more accessible, are often constrained from being displayed on 2D screens without specialized software, making it hard to fully appreciate the 3D nature of the trochlear groove. For example, a lateralized trochlea entry zone is an important indicator of lateral patellar tracking.^8^ This zone can be easily found by gliding a finger over a physical 3D model but can be more challenging to identify with 3D renderings on a 2D screen. The lateralization of this entry zone has been observed in both biological sexes, but prevalence values are poorly understood. It is known that females account for most patellofemoral instability (PFI) cases; therefore, sex could be an important consideration.

The purposes of this study were to examine a method to visualize a 3D rendered distal femur using 3D curvature analysis and to compare models of PFI with controls to study the reliability of the entry point to trochlear groove angle (EPTG) metric. We hypothesize that the reliability of the EPTG metric is significantly improved by adding 3D curvature analysis-based visualization of the distal femur.

## Methods

### Patient Selection

Patients seen by the senior author (J.P.F.) at our institution, an academic hospital system, between 2020 and 2023 were selected for the study. Inclusion criteria were patients with recurrent patellar instability with at least 2 patient-reported dislocation events, 16 years and older, and available high-resolution CT scans. CT-scans for the control cohort were acquired from the Free Access Decedent Database funded by the 10.13039/100005289National Institute of Justice grant number 2016-DN-BX- 0144 (New Mexico Descendent Image Database). Patients were age and sex matched with bilateral lower limb CT scans from the New Mexico Decedent Image Database (NMDID) to receive a nonsignificant difference in ratio between male versus female and age means. Exclusion criteria for the control group included a medical history of patellofemoral disease, including patellar instability as provided by the NMDID, and scan irregularities, such as metal artifacts, which would preclude the creation of clear 3D models. Age and sex were queried from the corresponding database and are representative of our patient population (i.e., primarily female and in their teens and 20s). The number of patients and controls was set to 30 each. Our institutional review board deemed this study exempt.

### Three-Dimensional Model Creation

CT scans were loaded into the commercially available segmentation software Simpleware ScanIP, and 3D models of the distal femur were created using the AS Ortho auto-segmentation tool. Segmentation was manually improved by one of the authors if needed. The model creation process is equivalent to the methods described by Beitler et al.^8^

### Curvature Visualization

The principal curvatures of the models were calculated using a Python algorithm utilizing the software package PyMeshLab.^9^ Principal curvatures consist of 2 values, one describing the direction and extent of the maximal curvature and the other describing the minimal curvature, which is perpendicular to the maximal curvature. For example, in a cylinder, the maximum curvature is tangential to its lateral sides and is valued at the inverse of its radius. The smaller the radius (i.e., tighter arc), the higher the curvature. Correspondingly, the minimal curvature is parallel to the symmetry axis and valued at zero, as there is no curvature along this direction. When a surface is bent outward (e.g., in the trochlea groove), that curvature becomes negative, with more negative values representing more curvature (e.g., “deeper” groove).

**Fig 1:**
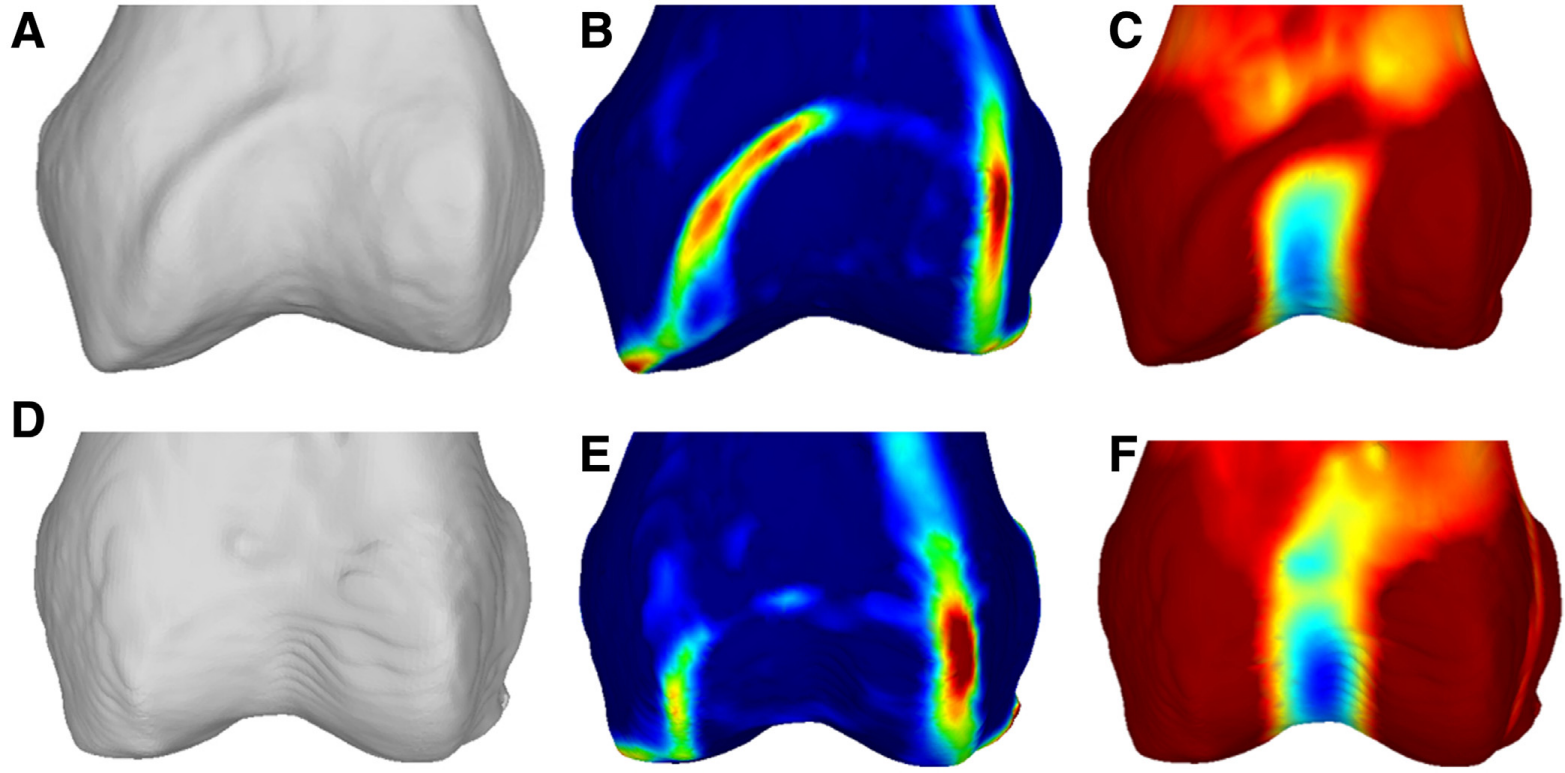
(A) Anteroposterior (AP) view of a 3-dimensional model created from a patellofemoral instability patient computed tomography scan. (B) AP view highlighting small structures with a high curvature (i.e., medial and proximal trochlear ridge). (C) AP view highlighting structures with a low curvature. (D-F) The corresponding AP views for a typical control. Typically, the proximal ridge in the patient cohort is more lateralized, and the trochlear groove terminates earlier, is lateralized, and is shallower.

Although curvature is a local parameter, it is possible to use different fitting parameters to calculate curvature at different scales at the femur (i.e., at smaller or larger scale, highlighting small [i.e., ridges] or large [i.e., trochlea groove] surface features). In the following, we utilize these 2 principal curvatures with different scale parameters to visualize the morphology of the distal femur (see Fig 1).

**Fig 2:**
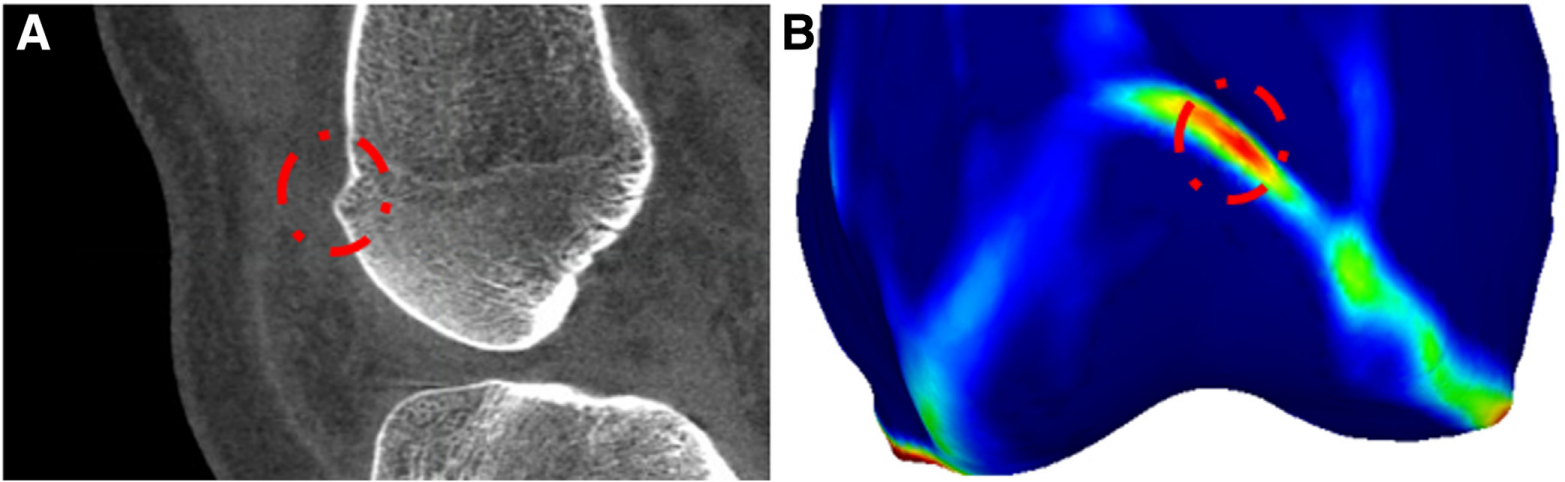
Highlighting of proximal trochlear ridges. (A) A computed tomography scan of a patient with a lateralized medial ridge. (B) The corresponding 3-dimensional model with curvature visualization. The trochlear ridge is highlighted in green to red, with cold colors indicating negative to low maximal curvature and warm colors indicating high curvature.

To highlight small, high curvature structures such as the ridges of the distal femur (see Fig 2), a fitting scale of 0.05 of condylar width was selected and the maximal curvature calculated. On average, for normal adult knees, this width is 78.0 ± 6.7 mm,^10^ leading to an expected fitting scale of 3.9 ± 0.3 mm. The calculated curvature maps were used to visualize regions of high small-scale curvature, such as the proximal trochlear ridges (see ridges in Fig 1 B and E).

**Fig 3:**
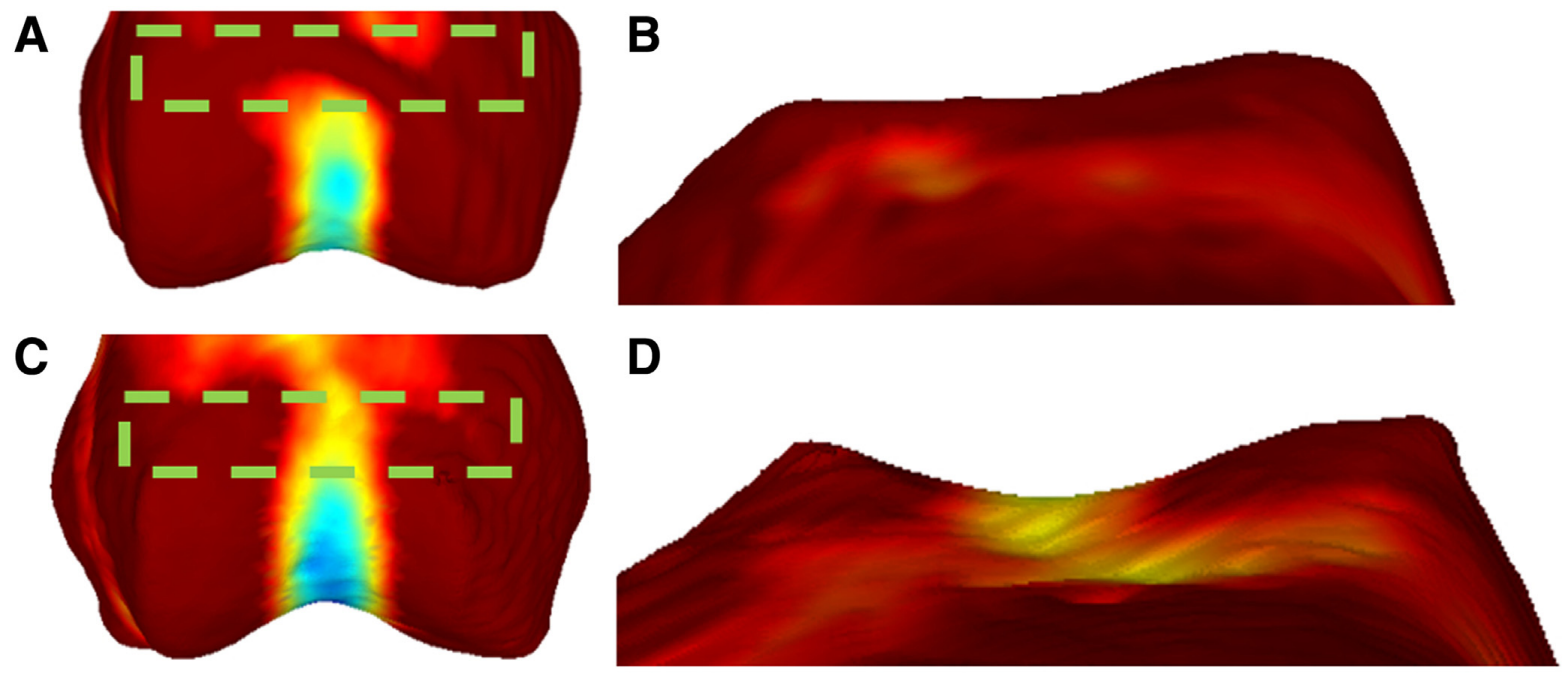
Highlighting trochlear groove curvature. (A) Minimal curvature overlaid on a patient’s distal femur. (B) The proximal trochlea seen from a superior view is flat/shallow, which is shown by the red coloring of the proximal trochlea in (A). (C) Typical distal femur of a control. The proximal trochlea is more curved, which can be seen in the cooler colors (C, D).

The distal femur is often modeled as a cylinder,^11^ meaning on a larger scale, the maximum curvature will be primarily perpendicular to its rotational axis and the minimal curvature along it, describing the curvature of the trochlear groove and the flanking condyles. Minimal curvature with a fitting factor of 0.13 of condylar width (10.1 ± 0.9 mm) was calculated and overlaid on the distal femur 3D models to highlight the trochlea groove and its curvature (see Fig 3).

The models with and without the curvature maps were used to create anteroposterior (AP) view screenshots (see Fig 1) for further evaluation. The screenshots and the algorithms used to create them are available in Appendix 1 (available at www.arthroscopyjournal.org) and the overlaid 3D models in the online repository.^12^

### EPTG Measurement

**Fig 4:**
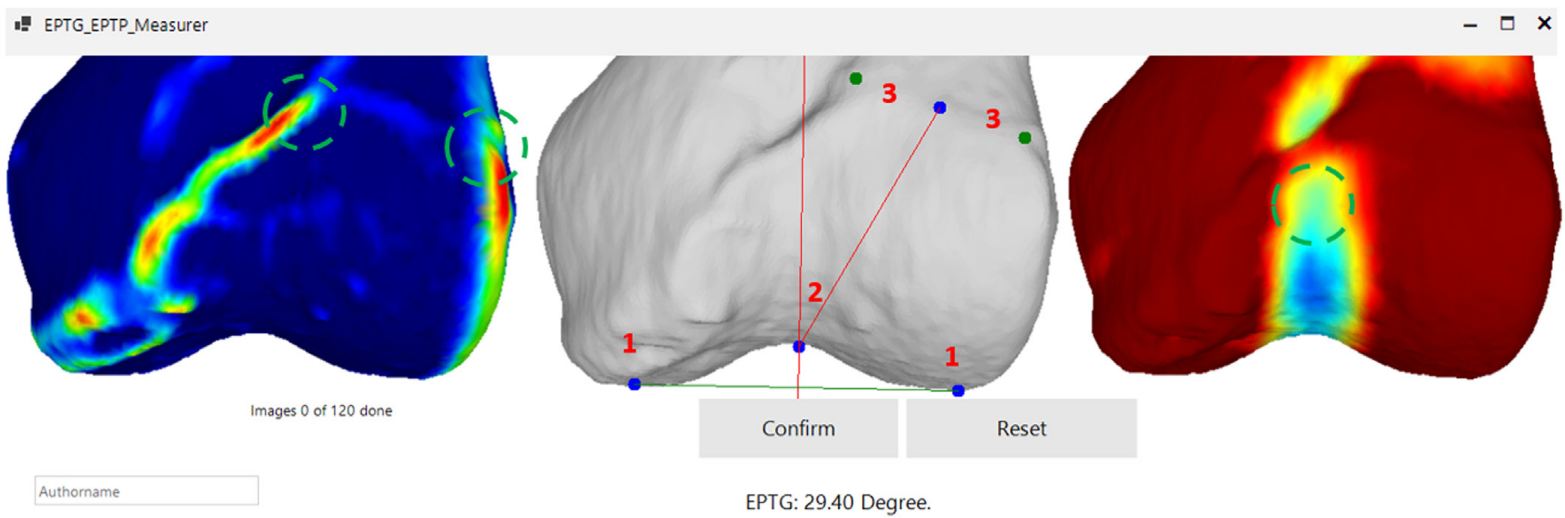
Custom tool, as seen by the raters, used to blind and allow them to conduct entry point to trochlear groove angle measurements. The authors select the condyles (1), the distal trochlear notch (2), and the medial and proximal ridge ends (3). The maximal curvature map (left) helps select the ridges, and the minimal curvature map (right) helps to determine the apex of the distal trochlear groove. Entry point to trochlear groove is the angle between the 2 straight red lines. The tool iterates in a randomized fashion through all 120 measurements.

The open-source EPTG measurement tool described by Beitler et al.^8^ was modified to include the curvature map images.^12^ The modified tool is included in the open-source repository.^13^ The screenshot data set for all 60 patients was loaded into the measurement tool, and 3 raters (J.P.F., J.M.S., and A.R.M.) measured the EPTG independently from each other in the following steps (see Fig 4) on the AP view without any curvature visualization.

The instructions in the program were as follows:

1. Click on the lateral and medial femoral condyles.
2. Click on the apex of the distal trochlear notch.
3. Click on the lateral end of the medial and the medial end of the lateral ridges (i.e., where the ridges flatten out and the curvature transitions from high to low).
4. Click on the transition point or select “there is no visible transition point.”

From these points, the measurement tool calculated the EPTG for each scan. To compare the reliability of measurements with and without the aid of the curvature maps, each AP view was duplicated, and the curvature maps were hidden for half of them. Measurements were taken in a randomized order for each rater. The 3 raters carried out all 120 measurements in a single sitting, creating a data set consisting of 60 EPTG measurements with and without curvature as a visual aid for each rater. Each measurement was recorded automatically via a screenshot for documentation. Rater 1 (J.M.S.) and rater 2 (A.R.M.) repeated the measurement sequence 3 times for intrarater calculation over a time period of 4 months.

### Qualitative Analysis

Curvature screenshots for patients and controls were displayed to the first author and the senior author. They evaluated qualitative differences in trochlea groove curvature in the minimal curvature maps and ridges in the maximal curvature maps between patients and controls.

### Statistical Analysis

A priori power analysis on significant differences between means of patients and controls was performed using descriptive data specified provided in the study introducing the EPTG^1^ (patients: 31.8° ± 10.3°; controls: 16.3° ± 9.4°), resulting in a minimal sample size of 9 per group. Power was assumed at 0.8. The study sample size was increased to 30 per group to reduce the range of the interclass correlation coefficient (ICC) 95% confidence interval calculation. Sex differences were not included in the power analysis and were therefore not statistically tested afterward.

Differences between age means in the patient and control groups were tested with a Mann-Whitney *U* test and differences in the sex ratio using a Fisher exact test. Significance was assumed at .05.

Differences between patients and controls were evaluated using a Mann-Whitney *U* test. Statistical significance was assumed at .05, adjusted with a Bonferroni-Holm correction. Inter-rater reliability of EPTG measurements with and without a curvature map aid was analyzed using 2-way random, single-score interclass correlation coefficients (ICC(A,1)) and evaluated following the existing literature.^14^ Intrarater reliability was calculated for 2 raters using 2-way mixed, single-score interclass correlation coefficients (ICC(A,1)), with each rater measuring the full data set thrice. Less than 0.5 was indicative of poor reliability, 0.5 to 0.75 was regarded as moderate, 0.75 to 0.9 was regarded as good, and larger than 0.9 was regarded as excellent reliability. Significance of difference between ICCs with and without the curvature map aid was analyzed with a Monte Carlo permutation test. The value of the ICC with aid was compared to the ICCs of 100,000 data sets created by randomly selecting either the measurement with or without aid for the 60 knees included in this study. The *P* value was calculated by taking the proportion of data sets with better ICC. Significance was assumed at .05.

## Results

Thirty patient knees and 30 control knees were segmented, their curvature calculated for visualization, and their AP views created for qualitative analysis (see Table 1 for demographics).

**Table 1:** Demographics, Means, Standard Deviations, and Inter- and Intra-Rater Reliability With Its Predicted Value and 95% Confidence Interval for the EPTG Measurement With and Without Curvature-Based Visual Aid Conducted in This Study

	Overall (n = 60)	Patients (n = 30)	Controls (n = 30)	*P* Value
Age, y	22.9 ± 7.1	23.9 ± 8.4	21.8 ± 5.6	.78
Sex, male/female	46/14	24/6	22/8	.76
EPTG	Mean ± SD			MWU *P* Value
With aid	22.9 ± 9.6	28.3 ± 6.7	17.4 ± 9.0	<.001
Without aid	23.7 ± 9.1	29.0 ± 5.4	18.4 ± 9.1	<.001
Female with aid	23.1 ± 9.5	27.8 ± 7.2	18.0 ± 9.2	NA
Male with aid	22.0 ± 10.3	30.6 ± 4.1	15.6 ± 8.6	
EPTG	ICC(A,1) [95% CI]—3 raters once			Reliability
With aid	0.87 [0.82-0.92]	0.78 [0.64-0.88]	0.85 [0.74-0.92]	Good to excellent
Without aid	0.64 [0.51-0.75]	0.45 [0.21-0.67]	0.56 [0.35-0.74]	Moderate to good
EPTG	ICC(C,1) [95% CI]—rater 1 thrice			Reliability
With aid	0.89 [0.84-0.93]	0.88 [0.80-0.94]	0.81 [0.69-0.90]	Good to excellent
Without aid	0.86 [0.80-0.91]	0.72 [0.56-0.85]	0.84 [0.74-0.92]	Good to excellent
EPTG	ICC(C,1) [95% CI]—rater 2 thrice			Reliability
With aid	0.91 [0.86-0.94]	0.93 [0.87-0.96]	0.83 [0.72-0.91]	Good to excellent
Without aid	0.78 [0.68-0.85]	0.58 [0.36-0.76]	0.76 [0.61-0.87]	Moderate to good

EPTG, entry point to trochlear groove angle; ICC, interclass correlation coefficient; MWU, Mann-Whitney *U* test; NA, not applicable.

The first and senior authors, a mechanical engineering PhD student and a fellowship-trained sports medicine orthopedist, found qualitatively that patients with patellofemoral instability had lateralized proximal ridges and trochlea grooves. The trochlear grooves were in general shallower and terminated in most cases before the trochlea ended proximally (as seen in Fig 1C). Controls generally had a funnel-like curvature proximal to the trochlea before transitioning into the groove. While these observations fit the general trend, each group had a small percentage of outliers (i.e., dysplastic patients with developed trochlear grooves [2/30 or 6.7%] and controls with short and shallow trochlear grooves and lateralized proximal ridges [2/30 or 6.7%]). All 60 screenshot triples (same as Fig 1) can be found in Appendix 1 (available at www.arthroscopyjournal.org).

The means, standard deviations, and ICCs for the EPTG measurements can be found in Table 1. The patient and control measurements were significantly (*P* < .001) different for EPTG with and without visual aid. Inter-rater ICCs for the measurement of the overall group with aids were significantly (*P* = .001) better compared to the measurements without aids using a Monte Carlo permutation test. Inter-rater reliability for measurements with aid was regarded as good to excellent compared to moderate to good for without. The screenshots of the conducted measurements can be found in the repository. Intrarater reliability showed rater dependency, with rater 1 not being significantly affected by the aids (*P* = .178) and rater 2 significantly affected (*P* = .015).

## Discussion

In this study, we found that the curvature-based visualization facilitates the understanding that the trochlear groove in recurrent PFI tends to be shallower or flat and lateralized proximally and that the medial ridge in patients with PFI tends to extend laterally into the trochlear groove. These observations are in line with studies conducted in 3D^1^^,^^2^^,^^8^^,^^15^^,^^16^ and 2D^3^^,^^4^ and should allow surgeons to better qualitatively understand the complex shape of the proximal trochlea instead of relying on a pure classification-based system. In addition, the trochlear groove appears to be shallower distally in those with PFI.

Additionally, we significantly (*P* = .016) improved the inter-rater reliability of the trochlea dysplasia metric EPTG from moderate-good to good-excellent reliability. We found that especially the measurements of the patient cohort benefited from the inclusion of the curvature-based visualization aids. The raters commented that including the visualization aids makes the measurement process faster and choosing the ends of the medial and lateral ridges is easier, making the measurement more reliable. The reliability of the EPTG measurement with visual aids is in line with or better than other trochlear dysplasia measures currently in use, most notably, the Dejour classification (Fleiss κ of 0.12 to 0.20),^5^ trochlea crossing sign (ICC, 0.46), sulcus angle (ICC, 0.34), trochlea depth (ICC, 0.74),^6^ and lateral trochlear inclination (ICC, 0.79).^16^

The inter-rater reliability improved significantly for rater 2 but not for the rater 1. Rater 1 was extensively involved in the development of the visualization aids and commented that they used what they learned from the visualization aids to interpret normal distal femur AP views, reducing the aid advantage given by the curvature map. Although rater 2 was also extensively trained in conducting these measurements, they did not see as many distal femora as rater 1. In our opinion, this indicates that the visualization aids based on curvature maps can be used for training physicians and students to better understand digital 3D models.

EPTG values for patients and controls with and without visualization aids are similar and in line with the initial study describing EPTG.^8^ Therefore, we conclude that adding the aids improves the reliability of the measurement without impairing the ability of the EPTG measurement to quantify the lateralization of the patella entry point. All 3D curvature maps in this study were algorithmically generated by the code provided open source within the code repository associated with this article. This enables others to freely use and modify it to run on their own 3D models. Because it is automatic, it adds minimal workload to anyone who already has the required 3D models. In our process, we utilized an auto-segmenter to create the 3D models, which required minor manual correction. In the future, we imagine that the combination of auto-segmentation and the curvature algorithm would allow the creation of 3D models with curvature visualization at the click of a button, facilitating the wide-scale application of this visualization in clinical decision-making.

### Limitations

The control group in this study was acquired from the NMDID; therefore, we cannot guarantee that all individuals with patellofemoral disease were excluded from the control group. However, the prevalence of such disease in this age group is low in the general population, and because inadvertent inclusion of such a knee would conservatively narrow the gap between the patient and control cohorts, we deem this limitation acceptable for this study.

In this study, we worked primarily with AP view screenshots taken from 3D models colored by curvature, limiting the information we could get to a single perspective and the understanding gained from the qualitative analysis. By rotating a colored 3D model in space and viewing it from additional angles, the surgeon would be able to gain additional information.

This study was not designed to establish sex differences in the general and patient population, even though there is currently a lack of such information in the literature. Therefore, due to the high likelihood of false-negative results, we did not conduct sex-dependent statistical tests in this study. Most of the scans used within the analysis were from female patients and controls; therefore, the results of this study might misrepresent the male patient population.

## Conclusions

Curvature-based visualization aids overlaid on a 3D model have the power to increase the information gained from 3D imaging and corresponding 3D models, amplifying their potential value in clinical decision-making. Such visualizations facilitate the identification of qualitative differences between patient and control morphology and improve the reliability of the EPTG trochlea dysplasia metric.

## Disclosures

All authors (J.M.S., N.P., A.R.M., S.T.D., K.B., C.M., S.M.T., D.H.W., J.P.F.) declare that they have no known competing financial interests or personal relationships that could have appeared to influence the work reported in this paper.
