# Supplementary material for: Visualization of Trochlear Dysplasia Using 3-Dimensional Curvature Analysis in Patients With Patellar Instability Facilitates Understanding and Improves the Reliability of the Entry Point to Trochlea Groove Angle
Source: Arthrosc Sports Med Rehabil. 2024 Sep 26;7(1):101010. doi: 10.1016/j.asmr.2024.101010 (PMC11873473; doi:10.1016/j.asmr.2024.101010)

PFI Patients 1-30

Supplement 1 for: Visualization Tool for Trochlear Dysplasia using 3D Curvature Analysis Facilitates Understanding and Improves the Reliability of the Entry-Point to Trochlea Groove Angle (EPTG)

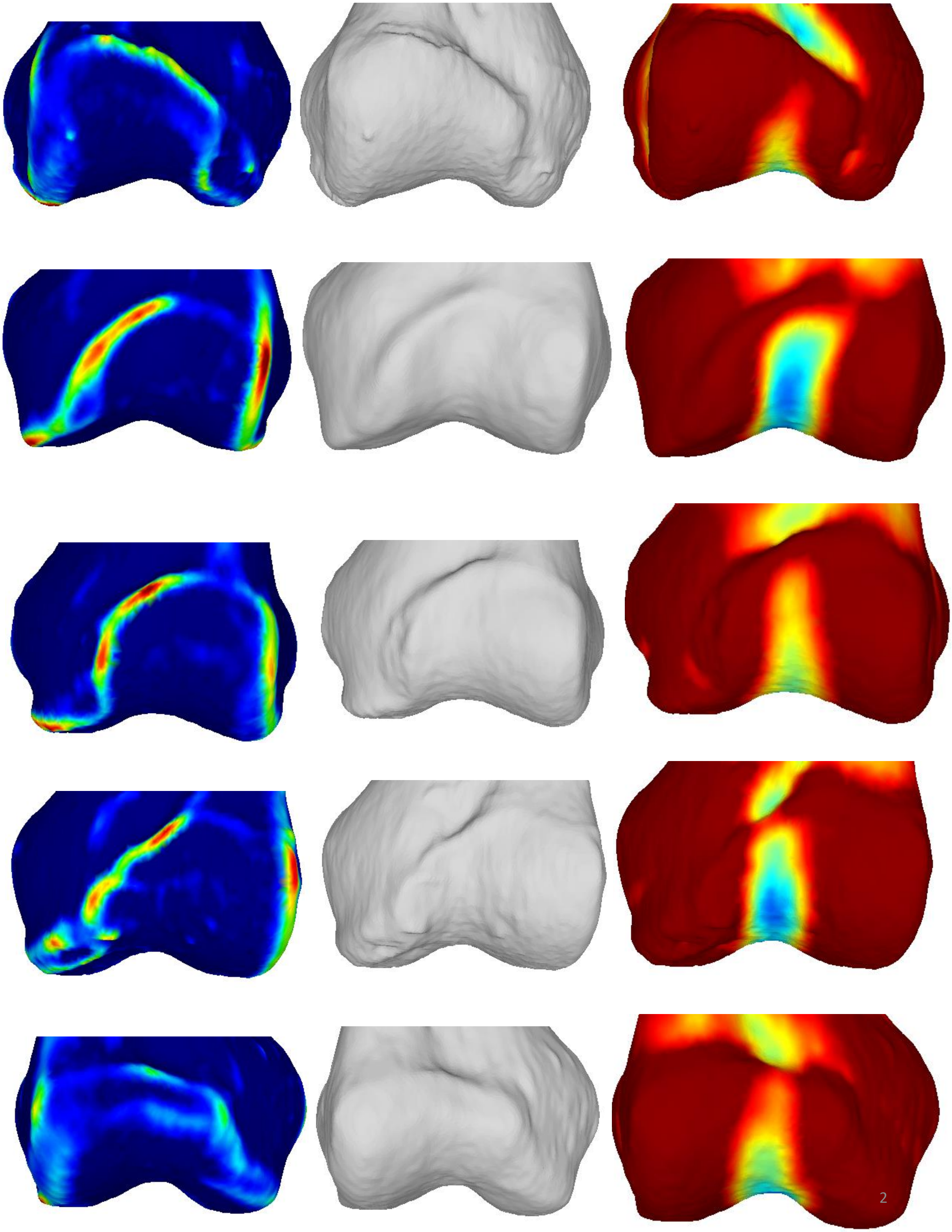

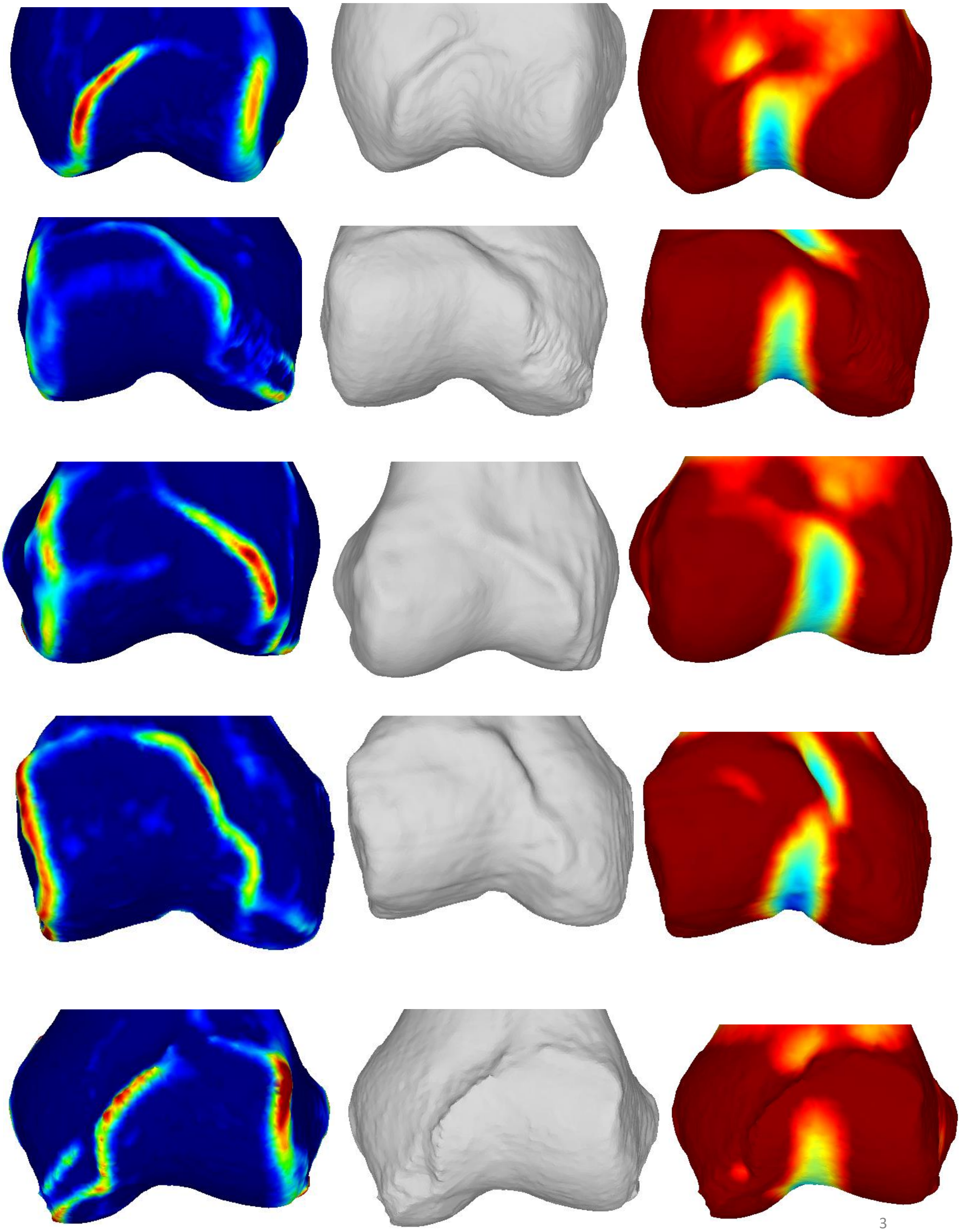

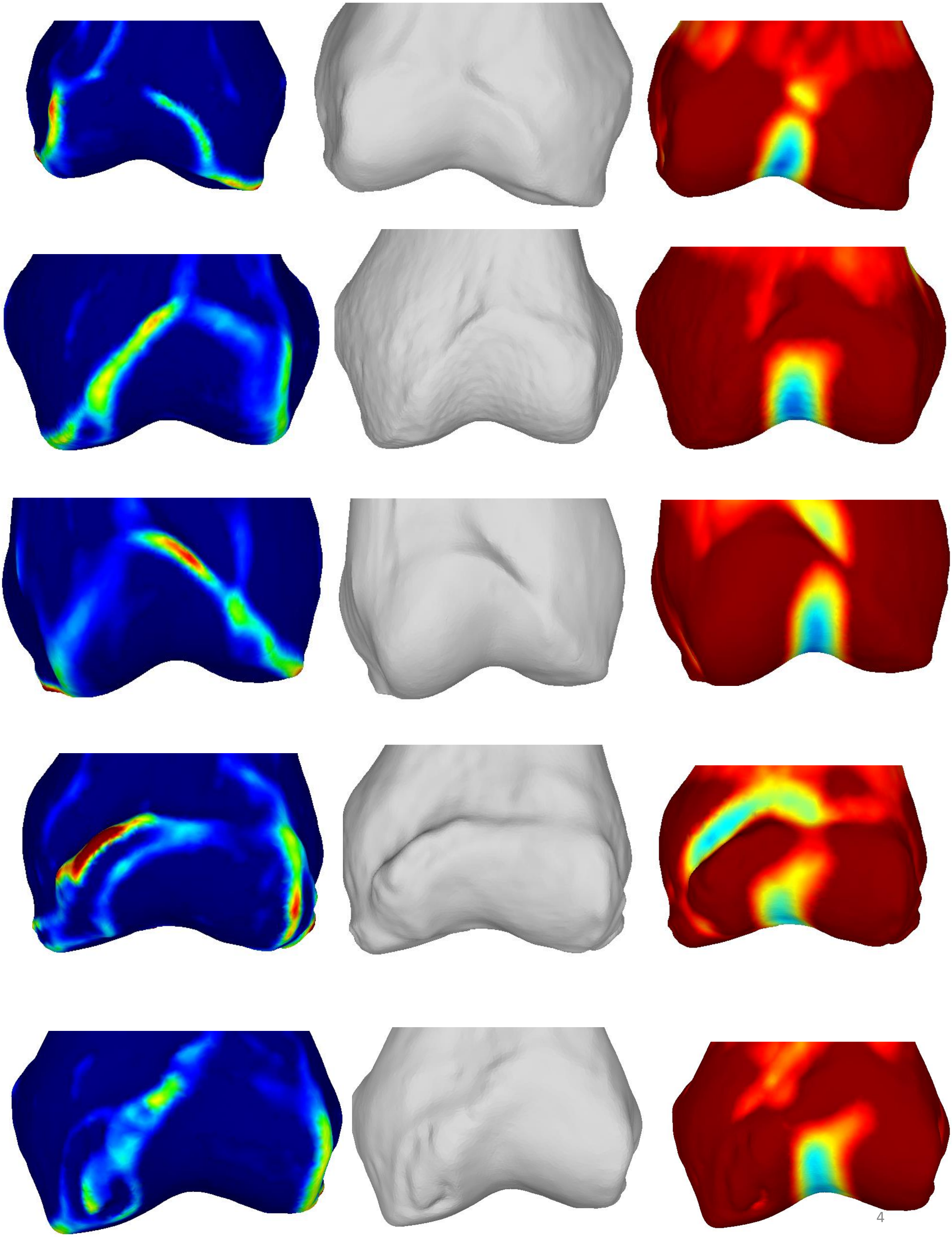

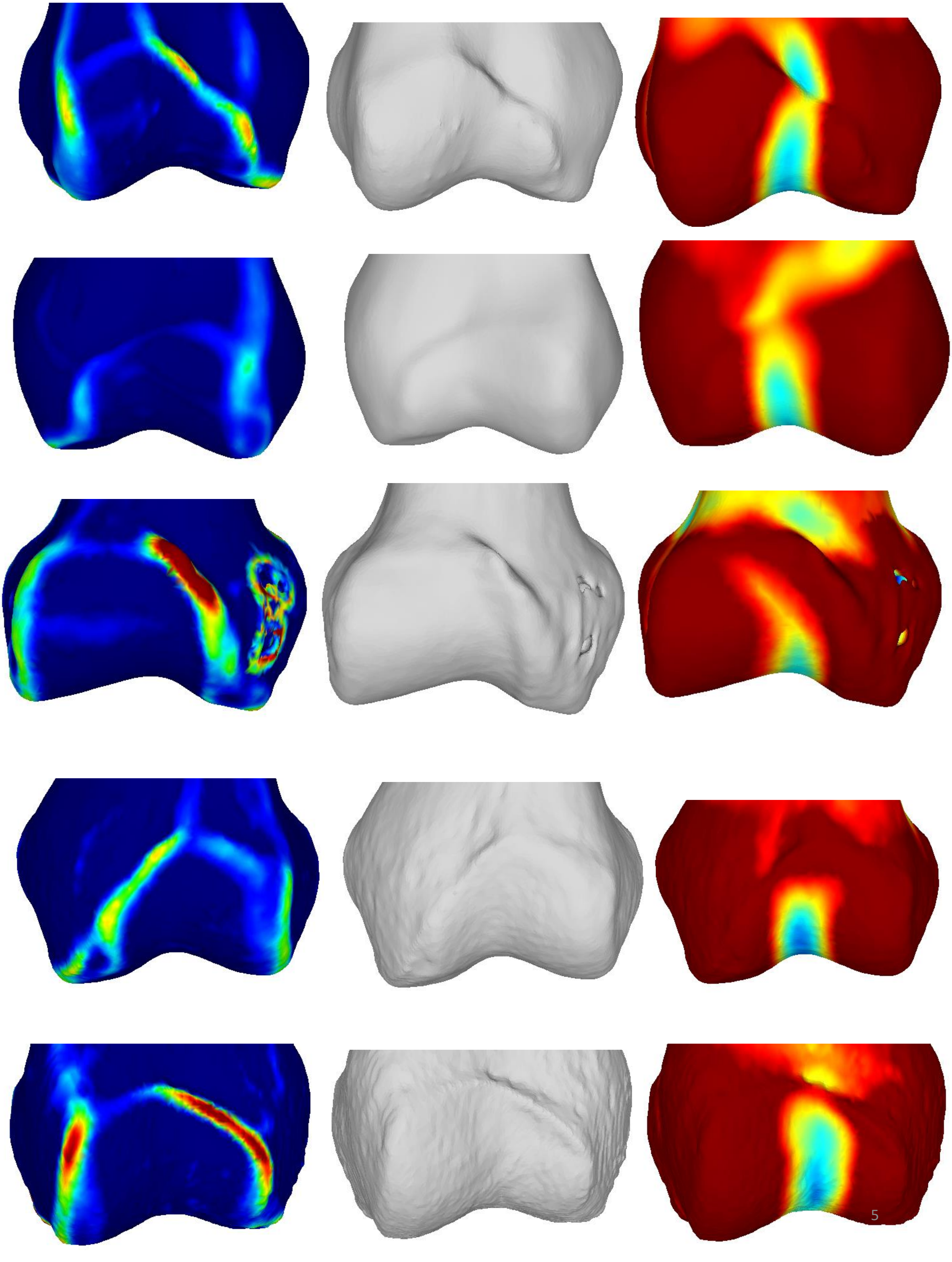

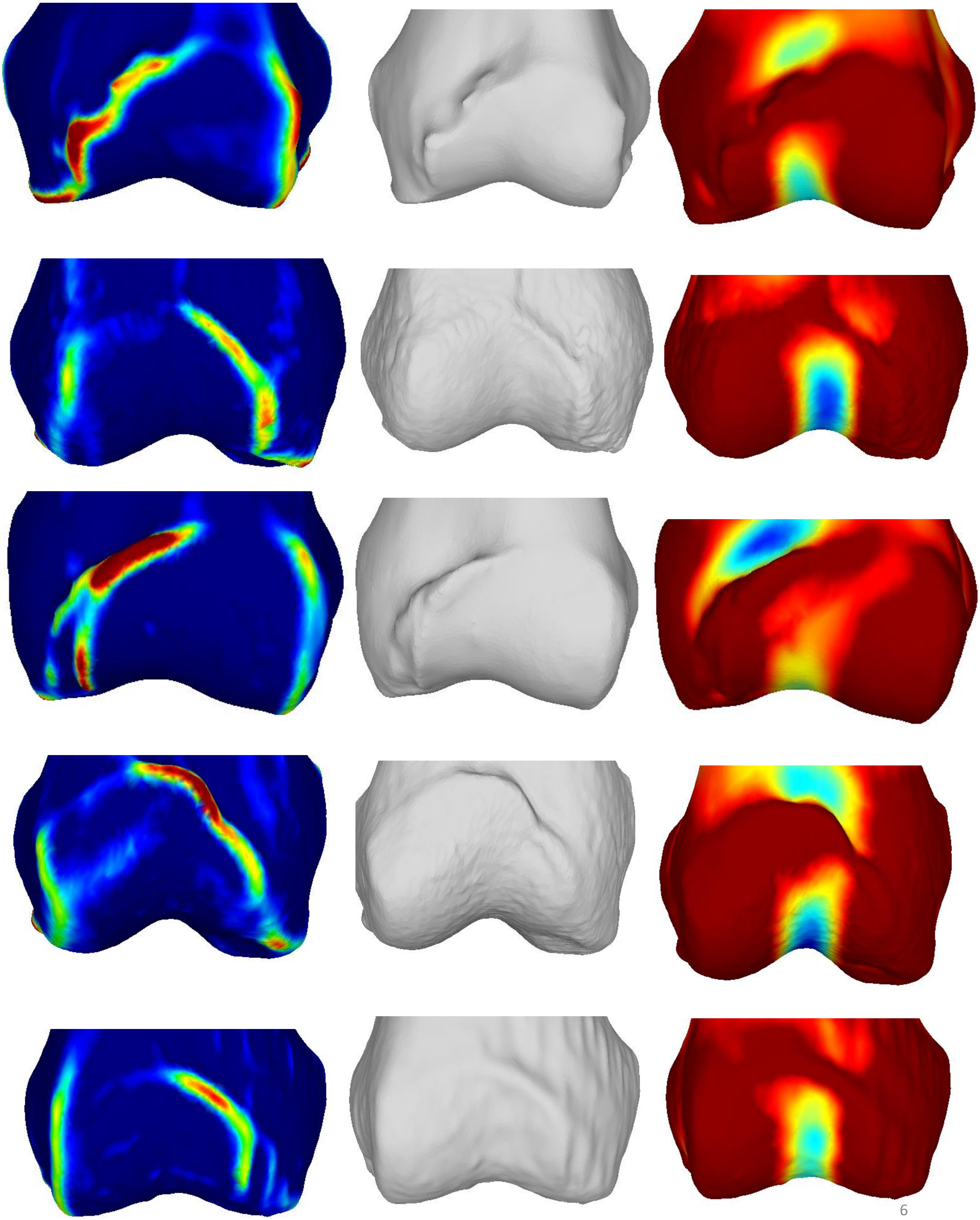

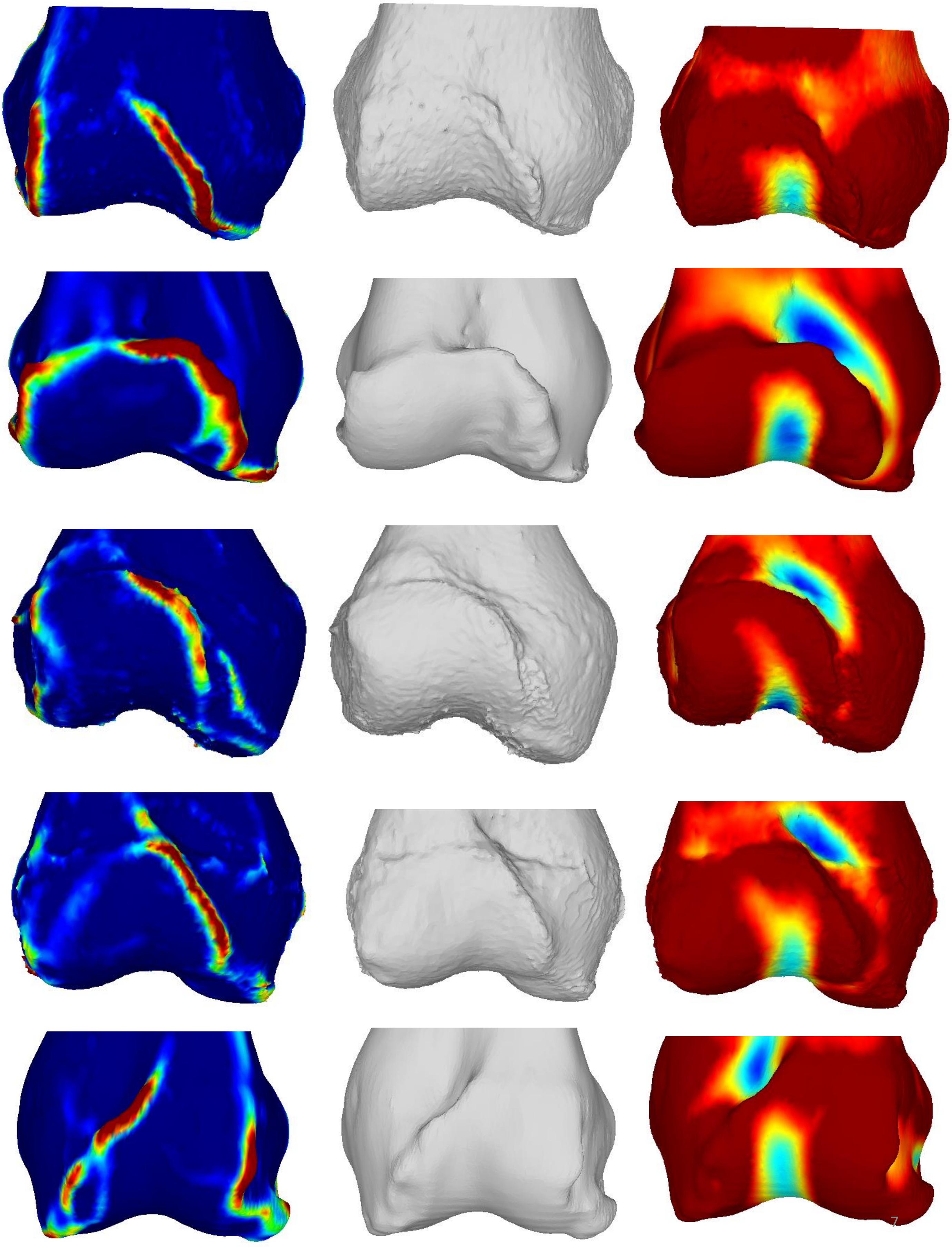

Controls 1-30

Supplement 1 for: Visualization Tool for Trochlear Dysplasia using 3D Curvature Analysis Facilitates Understanding and Improves the Reliability of the Entry-Point to Trochlea Groove Angle (EPTG)

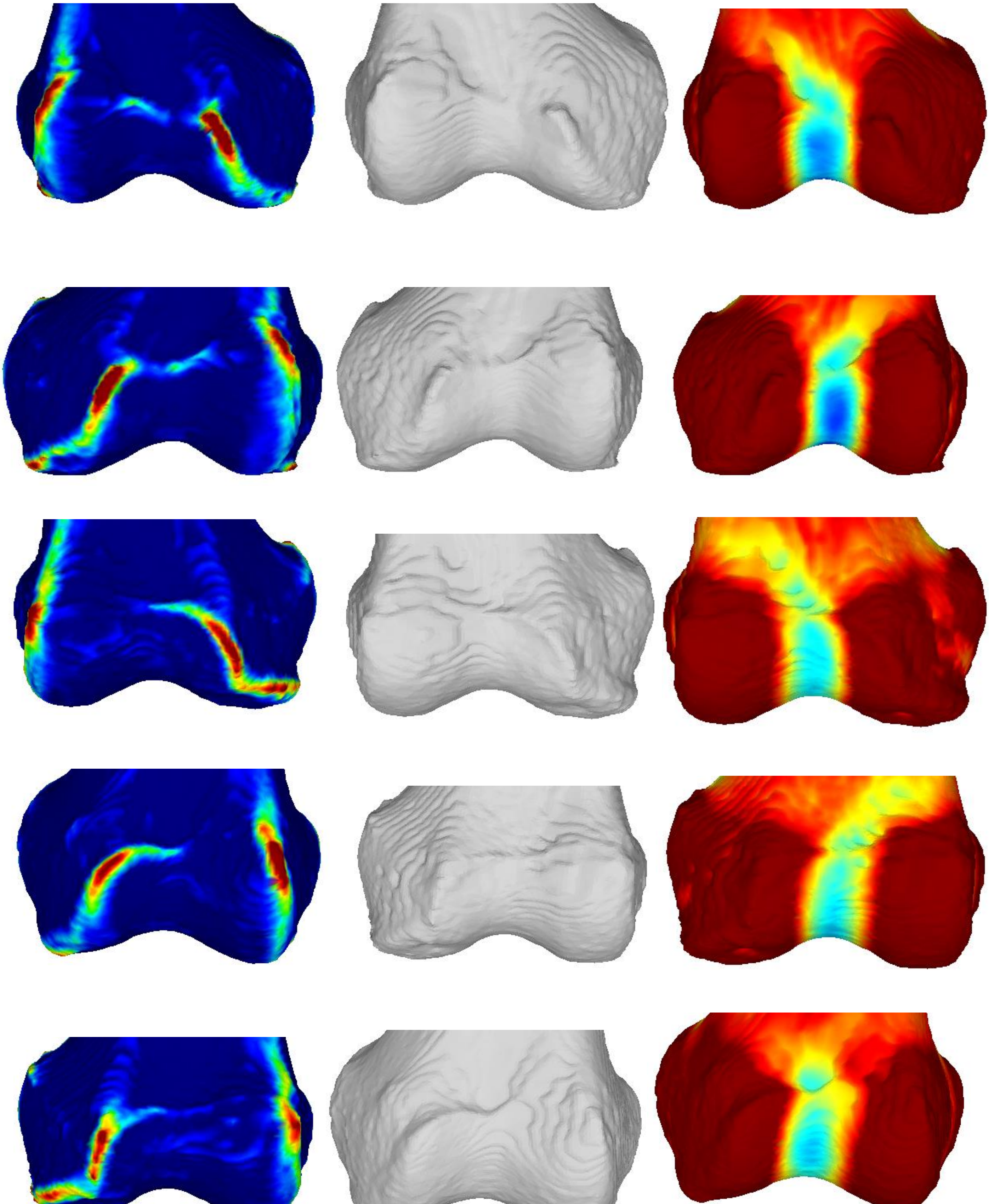

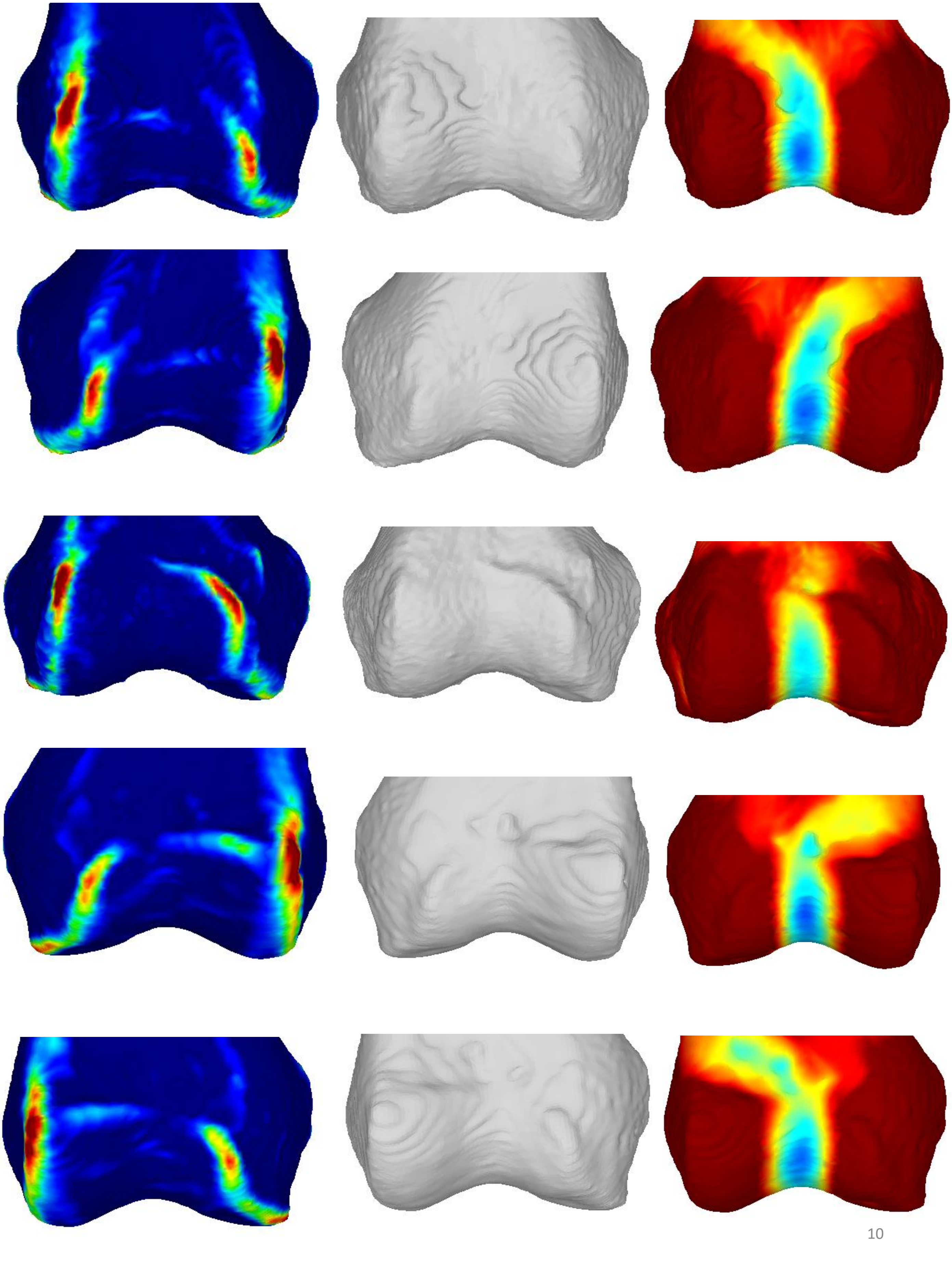

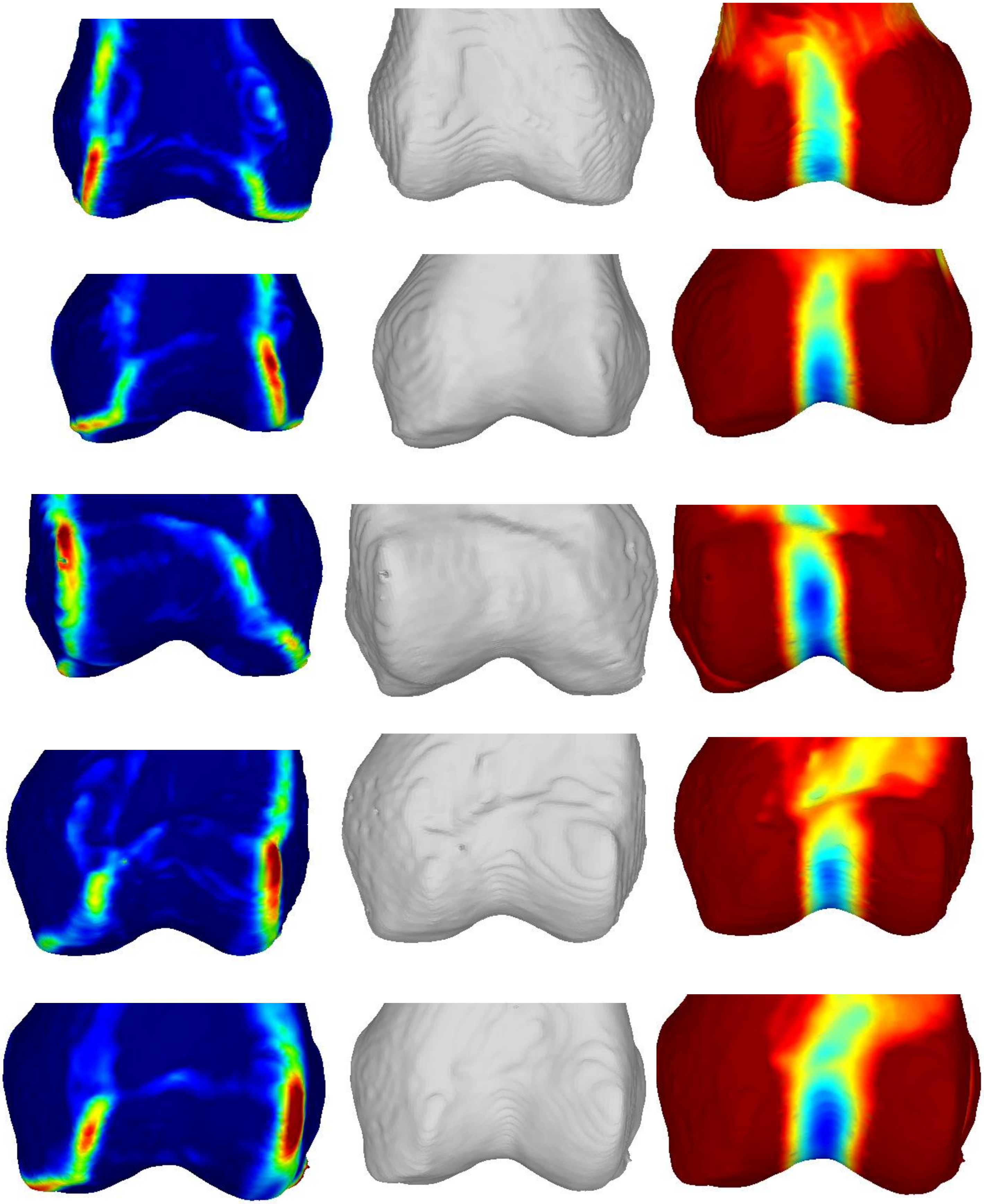

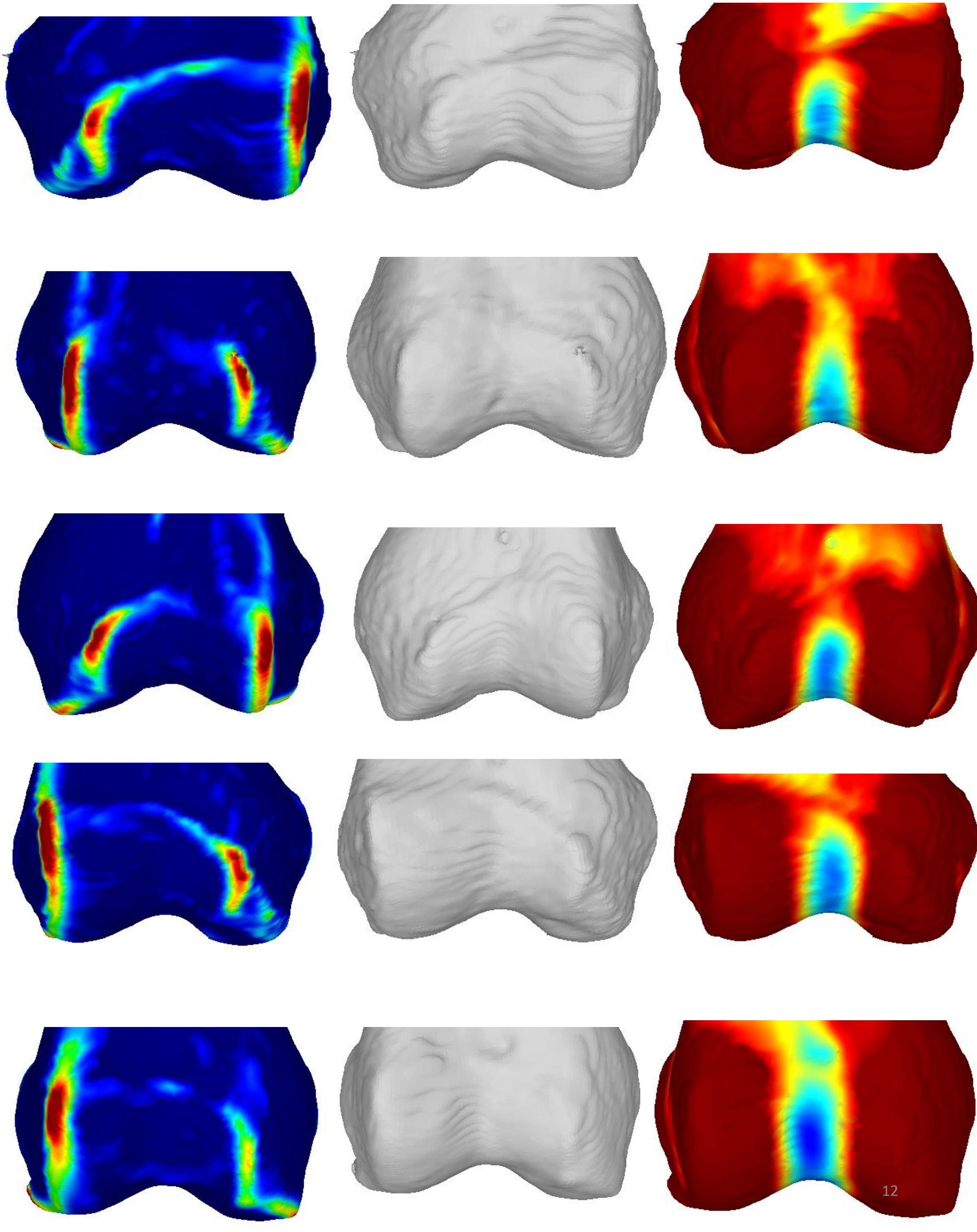

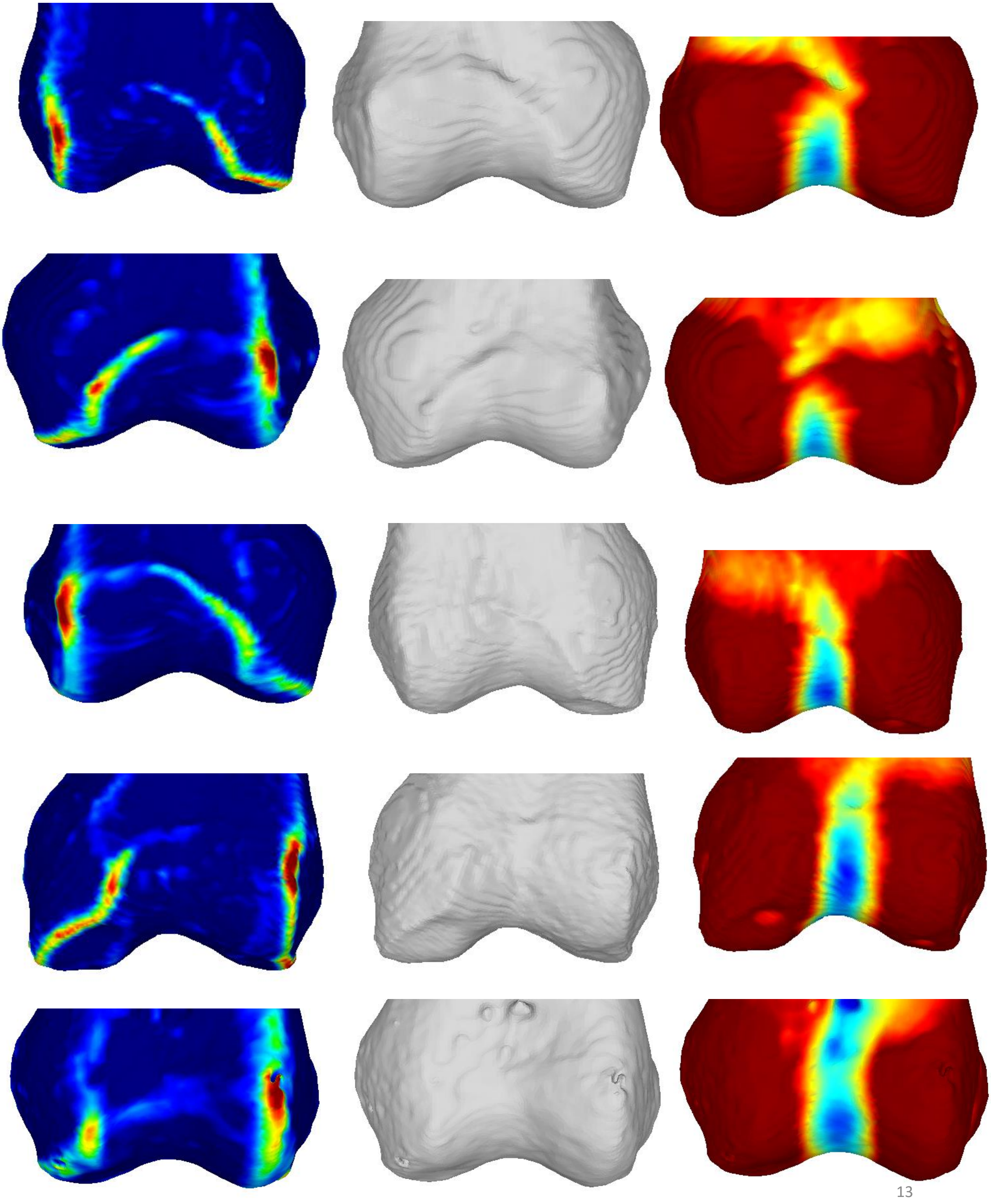

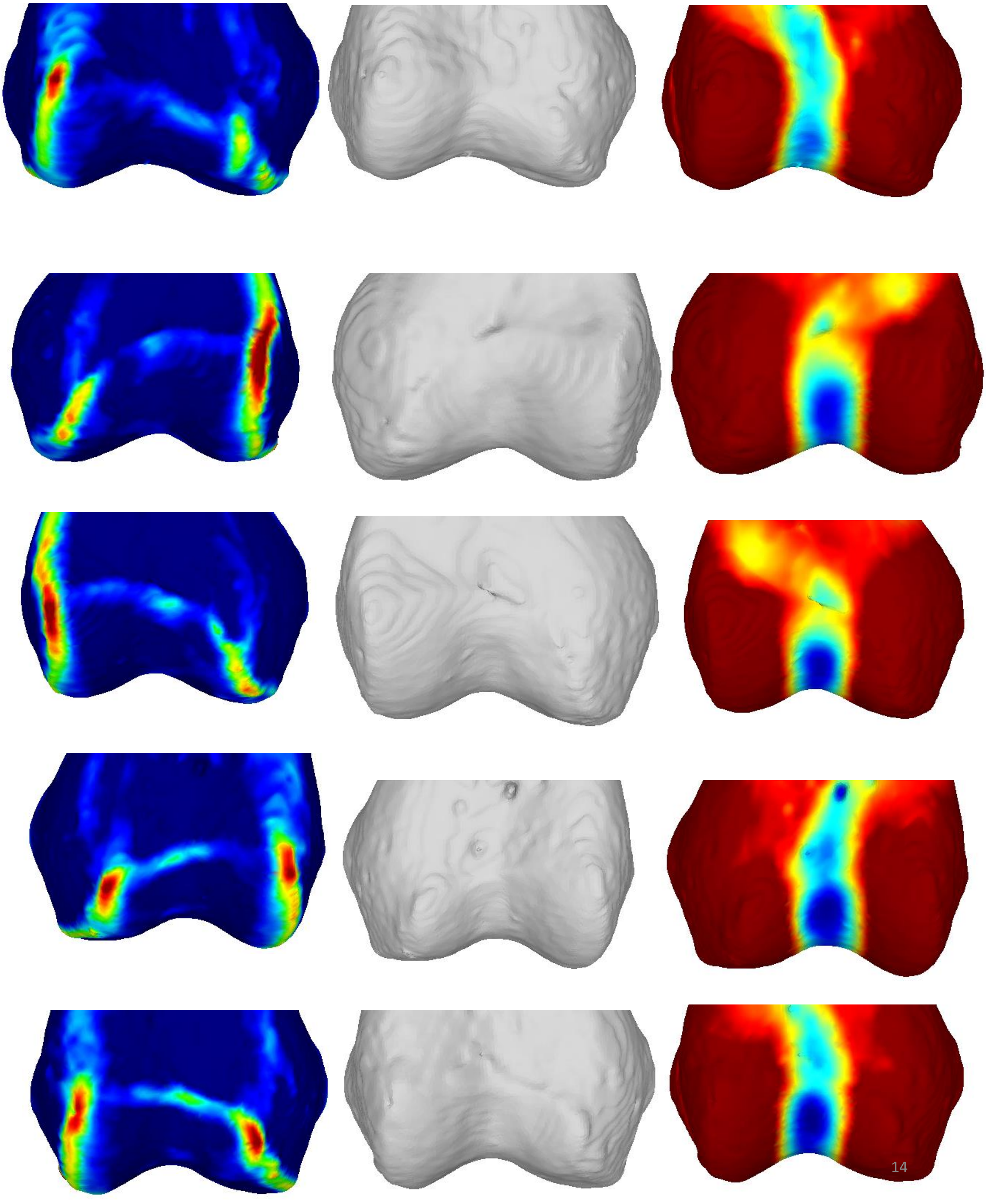

Supplement: Supplemental Data [file mmc1.pdf]
